# Almond Consumption and Processing Affects the Composition of the Gastrointestinal Microbiota of Healthy Adult Men and Women: A Randomized Controlled Trial

**DOI:** 10.3390/nu10020126

**Published:** 2018-01-26

**Authors:** Hannah D. Holscher, Andrew M. Taylor, Kelly S. Swanson, Janet A. Novotny, David J. Baer

**Affiliations:** 1 Department of Food Science and Human Nutrition, University of Illinois, Urbana, IL 61801, USA; amtaylr3@illinois.edu; 2 Division of Nutritional Sciences, University of Illinois, Urbana, IL 61801, USA; ksswanso@illinois.edu; 3 Department of Animal Sciences, University of Illinois, Urbana, IL 61801, USA; 4 USDA, ARS, Beltsville Human Nutrition Research Center, Beltsville, MD 20705, USA; Janet.Novotny@ARS.USDA.GOV (J.A.N.); David.Baer@ARS.USDA.GOV (D.J.B.)

**Keywords:** nuts, microbiome, fiber, fat, fermentation

## Abstract

Background: Almond processing has been shown to differentially impact metabolizable energy; however, the effect of food form on the gastrointestinal microbiota is under-investigated. Objective: We aimed to assess the interrelationship of almond consumption and processing on the gastrointestinal microbiota. Design: A controlled-feeding, randomized, five-period, crossover study with washouts between diet periods was conducted in healthy adults (*n* = 18). Treatments included: (1) zero servings/day of almonds (control); (2) 1.5 servings (42 g)/day of whole almonds; (3) 1.5 servings/day of whole, roasted almonds; (4) 1.5 servings/day of roasted, chopped almonds; and (5) 1.5 servings/day of almond butter. Fecal samples were collected at the end of each three-week diet period. Results: Almond consumption increased the relative abundances of *Lachnospira*, *Roseburia*, and *Dialister* (*p* ≤ 0.05). Comparisons between control and the four almond treatments revealed that chopped almonds increased *Lachnospira*, *Roseburia*, and *Oscillospira* compared to control (*p* < 0.05), while whole almonds increased *Dialister* compared to control (*p* = 0.007). There were no differences between almond butter and control. Conclusions: These results reveal that almond consumption induced changes in the microbial community composition of the human gastrointestinal microbiota. Furthermore, the degree of almond processing (e.g., roasting, chopping, and grinding into butter) differentially impacted the relative abundances of bacterial genera.

## 1. Introduction

Diet is a contributing factor to the development of numerous diseases including obesity, cardiovascular disease, and type 2 diabetes. Increasingly, these complex metabolic diseases are also being associated with the gastrointestinal microbiota [1]. The gastrointestinal microbiota, an ecosystem containing trillions of microorganisms, including bacteria, archaea, and fungi, is, in essence, a microbial organ that possesses more than 150 times more genes than the human genome [2]. Importantly, these microbes possess genes that allow for the metabolism of foods that escape digestion by human alimentary enzymes, thereby aligning diet as an important modulator of the interrelationship between diet, the gastrointestinal microbiota, and host health. 

Diet is a key modulator of the human gastrointestinal microbiota—habitual dietary patterns [3], rapid changes in dietary macronutrient composition [4], eating behaviors [5], and increased intake of different types of plant-based foods [6] have been linked to the composition of human gastrointestinal microbiota. To date, much of the research on specific diet components has focused on the impact of consumption of different dietary fibers and prebiotics on the human gastrointestinal microbiota [7,8,9,10]. These studies have revealed that the molecular composition, including the glycosidic linkages between monosaccharides, as well as physicochemical properties of fibers influence the microbial composition and production of bacterial metabolites including short-chain fatty acids [11]. Preclinical research and in vitro studies have also demonstrated that dietary fats can differentially impact the gut microbiota and that certain microbes can utilize fatty acids as energy substrates [12,13,14,15]. Almonds are a nutrient-dense food that provide a good source of dietary fiber (5 g per 42 g almonds) as well as unsaturated fatty acids (20 g per 42 g almonds).

Dietary consumption of nuts is associated with reduced risk of diseases including, obesity, cardiovascular disease, type 2 diabetes, and cancer [16,17]. Furthermore, epidemiological evidence indicates that individuals who regularly consume tree nuts exhibit lower waist circumference and superior metabolic profiles [18]. Interestingly, clinical trials with almonds have demonstrated that food form (e.g., whole, chopped, ground) differentially impacts metabolizable energy due to incomplete absorption of macronutrients in the gastrointestinal tract [19]. As nutrients that are not absorbed in the proximal gastrointestinal tract are subject to microbial fermentation, dietary consumption of nuts in different forms stand to influence the composition of the gastrointestinal microbiota. Indeed, the health-related effects of nut consumption may not only be related to their metabolizable energy, but also their impact on the gastrointestinal microbiota. One hypothesized connection between the metabolic improvements shown with almond consumption is increased butyrate production by gastrointestinal microbes. In vitro, microbial fermentation of finely ground almonds has been shown to increase butyrate concentrations [20], and preclinical studies have demonstrated that increased butyrate concentrations are linked to reduced gut inflammation [21], improved gut barrier function [22], and glucose tolerance [23]. 

Previously, we conducted a controlled-feeding, randomized, cross-over trial in healthy adult men and women to determine the metabolizable energy of different forms of almonds, including whole, natural almonds; whole, roasted almonds; roasted, chopped almonds; and almond butter and reported that the metabolizable energy of whole natural almonds, whole roasted almonds, and chopped almonds was up to 25% lower than predicted by calculation using the Atwater values [19]. Indeed, the process of roasting and chopping almonds reduced their hardness and particle size, increasing the digestion and absorption of lipids in the small intestine, which resulted in metabolizable energy values of 4.42 kcal/g for whole natural, 4.86 kcal/g for whole roasted, and 5.04 kcal/g for roasted chopped almonds. The addition of pureeing the roasted almonds into butter ablated this inhibition of digestion and absorption revealing that the calculated and measured metabolizable energy of almond butter was not different, 6.68 kcal/g and 6.53 kcal/g, respectively. Thus, the degree of almond processing (e.g., roasting, chopping, and grinding) affected the nutrients delivered to the large intestine and excreted in the feces. In this follow-up report, we aimed to assess our secondary outcomes of the study and determine the effect of almond consumption and processing on the gastrointestinal microbiota by subjecting the same fecal samples from the clinical trial on metabolizable energy of almonds to high-throughput sequencing. The objectives of the current study were to determine how almond consumption and almond form (e.g., whole, whole roasted, chopped, and butter) differentially impact the bacteria, archaea, and fungi in the human gastrointestinal tract. We hypothesized that consumption of almonds and food form would impact the human fecal microbiota compared to control.

## 2. Methods

### 2.1. Experimental Design, Treatments, and Subjects

This report is a follow-up to a previously conducted clinical trial (NCT02034383). Details of the design and results of the primary study, which assessed the metabolizable energy of almonds, were previously reported [19]. Herein, secondary outcomes related to almond consumption on the gastrointestinal microbiota were assessed. Briefly, this was a controlled-feeding, randomized, cross-over study with five three-week diet periods, each diet period was separated by a one-week non-controlled diet break (wash-out) period. The controlled diets, fed at weight maintenance, consisted of a base diet designed to represent the typical American diet, with a macronutrient composition of 53% of kcals from carbohydrates, 32% of kcals from fat, and 15% of kcals from protein, and 0 g/day of almonds. All foods were identical between the control and almond diets, except for the almonds. During the four almond diet periods of the study, the controlled diet was scaled down to allow for the isocaloric inclusion of 42 g/day of varying forms of almonds: whole, natural almonds; whole, dry roasted almonds; chopped, dry roasted almonds; and dry roasted, almond butter. 

Study participants were recruited in the Washington, DC metropolitan area surrounding the Beltsville Human Nutrition Research Center (BHNRC) in Beltsville, MD, USA. All subjects gave their informed consent for inclusion before participating in the study. The study was conducted in accordance with the Declaration of Helsinki, and the protocol and informed consent document were reviewed and approved by the Medstar Health Research Institute (Protocol #2013-136). Participants were excluded if they were <25 or >75 years of age, had a body mass index (BMI) <20 or >38 kg/m^2^, blood pressure ≥160/100 mm Hg, fasting blood glucose ≥126 mg/dL, fasting total blood cholesterol ≥280 mg/dL, and fasting triglycerides ≥300 mg/dL. Participants were excluded if they had any of the following diseases or conditions: allergy to almonds or other nuts, gastrointestinal disease, malabsorptive syndromes, liver disease, pancreatic disease, kidney disease, gout, hyperthyroidism, untreated or unstable hypothyroidism, certain cancers, or type II diabetes; history of bariatric or certain other surgeries related to weight control; history of eating disorders or other dietary patterns which are not consistent with the dietary intervention (e.g., vegetarians, very low fat diets, high protein diets); greater than 10% change in body weight within the last 12 months; pregnant women or women who plan on becoming pregnant during the study period; women who had given birth within the past 12 months; and tobacco users. A random number generator was utilized for study participant randomization following the creation of a randomization scheme that stratified the 18 participants (10 males; eight females) by sex and BMI. Each participant was assigned to a treatment sequence that consisted of each of the five diet periods.

### 2.2. Sample Collection and Analysis

Fecal samples were collected at the end of each of the five diet treatment periods (e.g., control, whole almonds, whole roasted almonds, roasted chopped almonds, and almond butter) for microbiota analyses. Following fecal collection and homogenization, samples were stored at −80 °C at the BHNRC prior to overnight shipment on dry ice to the University of Illinois and subsequent storage of samples at −80 °C until analysis. Fecal DNA was extracted according to manufacturer’s guidelines using the MoBio Powerlyzer Powersoil Kit (MoBIO Laboratories Inc., Carlsbad, CA, USA). After extraction, sequencing was performed at the W. M. Keck Center for Biotechnology at the University of Illinois as previously described [24]. Briefly, bacterial (V4 region of 16S rRNA; 515F/806R: 5′-GTGCCAGCMGCCGCGGTAA, 5′-GGACTACHVGGGTWTCTAAT), archaeal (ArchaeaF/ArchaeaR: 5′-GYGCASCAGKCGMGAAW, 5′-GGACTACVSGGGTATCTAAT) and fungal (ITS1F/ITS4R: 5′-TTCGTAGGTGAACCTGCGG, 5′-TCCTCCGCTTATTGATATGC) genes were amplified using a Fluidigm access array (Fluidigm, South San Francisco, CA, USA). Following amplification, a fragment analyzer (Advanced Analytics, Ames, IA, USA) was utilized to check the quality of the amplicon pool and confirm amplicon region and size. Next, a DNA pool was generated by combining equimolar amounts of the amplicon from each sample. Then, amplicon pools were size-selected on a 2% agarose E-gel (Life Technologics, Grand Island, NY, USA) to remove low molecular weight DNA fragments. A Qiagen gel purification kit (Qiagen, Valencia, CA, USA) was used to extract the amplicon pools from the gel and remove impurities. Then, size-selected, cleaned amplicon pools were analyzed using an Agilent Bioanalyzer (Advanced Analytics, Ames, IA, USA) to confirm size selection and quality. Lastly, the amplicon pool was sequenced using an Illumina MiSeq (Illumina Inc., San Diego, CA, USA) with version 3 chemistry. 

Sequence data derived from the sequencing process were trimmed using the the open access FASTX-toolkit (v 0.0.14), and demultiplexed and analyzed with the open access bioinformatics software QIIME 1.8.0. Briefly, bacterial and archaeal sequences were clustered into operational taxonomic units (OTUs) using the default UCLUST closed-reference OTU picking algorithm (pick_closed_reference_otus.py) against the curated Greengenes 13_8 reference OTU database (97% similarity threshold), which discards sequences that do not match to the reference database [25,26]. Fungal OTUs were generated using the open reference OTU picking algorithm (pick_open_reference_otus.py) against the UNITE OTUs ITS 12_11 reference database [27]. Singletons (OTUs observed fewer than two times) and OTUs that represented less than 0.005% of the total observations were removed [28] before rarefying the bacterial sequences to an even sampling depth of 8227 sequences per sample to assess alpha (observed OTUs and phylogenetic diversity) and beta diversity (weighted and unweighted Unifrac distances) measures as well as the impact of treatment on OTU abundances that that represented ≥0.5% of the total sequences. 

### 2.3. Statistics

Sequence percentages were analyzed using the mixed models procedure of SAS (version 9.4; SAS Institute, Inc., Cary, NC, USA) with treatment as a fixed effect and participant and period as random effects. Post hoc Dunnets adjustments were used to control for multiple comparisons. To determine if the presence or absence of almonds affected the fecal microbiota, contrast statements were utilized to compare almond (pooled whole, whole roasted, chopped, and butter) to the control diet period. To assess the effect of processing on the microbiota, each almond group (e.g., whole, whole roasted, chopped, or butter) was contrasted against control. The UNIVARIATE procedure and Shapiro–Wilk statistic were used to test for data normality, and log transformations were utilized as needed. The Wilcoxon test was used for fungal count analyses. A probability of *p* ≤ 0.05 was accepted as statistically significant, and *p* ≤ 0.10 was considered a trend.

## 3. Results

All 18 participants, 10 men and eight women, that were randomized completed the study. Participants had a mean BMI of 29.7 + 4.4 kg/m^2^ and an age of 56.7 + 10.2 years (Table 1). 

Following demultiplexing and quality filtering, the mean and standard deviation of bacterial OTUs identified using closed reference OTU picking was 33,194 ± 9686, with a minimum of 8227 counts per sample. As such, all samples were rarefied to an even sequencing depth of 8227 for subsequent analyses. At the phyla level, more than 90% of bacterial sequences were dominated by Firmicutes (64.2%) and Bacteroidetes (27.5%), with smaller proportions of Actinobacteria (3.8%), Proteobacteria (2.0%) and Verrucomicrobia (1.6%). Forty bacterial families were identified. The top seven families, Ruminococcaceae (28.9%), Lachnospiraceae (20.4%), Bacteroidaceae (21.0%), an undefined family in the Clostridales order (8.1%), Veillonellacaea (3.3%), Bifodobacteriaceae (2.5%), and Verrucomicrobiaceae (1.6%), comprised approximately 90% of the sequences. Seventy-eight genera were classified with 21 representing greater than 90% of total sequences and 34 representing <1%; the top 10 genera, *Bacteroides*, unclassified Ruminococcaceae, unclassified Clostridiales, an unclassified Lachnospiraceae, *Coprococcus*, *Faecalibacterium*, *Ruminococcus*, *Bifidobacterium*, *Blautia*, and an unclassified genera in the Rikenellacaeae family represented approximately 77% of total sequences classified at the genera level. 

Archaeal sequences accounted for approximated 8.4% of total sequences. Following demultiplexing and quality filtering, the mean and standard deviation of archaeal OTUs identified using closed reference OTU picking was 3081 ± 5289, with a maximum of 26,030 counts per sample and a minimum of zero counts per sample. Treatment did not impact archaea relative abundances. Fungi accounted for approximately 0.1% of total sequences; the mean and standard deviation was 256 + 560 OTUs per sample. The minimum was one OTU/sample (12 individuals), and the maximum was 2410 sequences. Study participants had more (*p* < 0.05) fungal OTUs when consuming the whole almonds treatment compared to the other treatment groups. 

To determine if the presence or absence of almonds in the diet affected the fecal microbiota, contrast statements were utilized to compare almond (pooled whole, whole roasted, chopped, and butter) to the control diet period. The data revealed that almond consumption decreased the relative abundance of the Actinobacteria phylum, as well as *Bifidobacterium* and *Parabacteroides* (*p* < 0.05). The relative abundance of *Lachnospira*, *Roseburia*, *Clostridium*, and *Dialister* (*p* ≤ 0.05) increased when the almonds were consumed compared to the control (Table 2). To assess the effect that processing has on the microbiota, each almond group (e.g., whole, whole roasted, chopped, or butter) was contrasted against the control. These results revealed that processing differentially impacted the microbiota. Compared to the control, chopped almonds increased the relative abundance of *Lachnospira*, *Roseburia*, and *Oscillospira* (*p* < 0.05); whole roasted almonds increased (*p* = 0.03) the relative abundance of *Lachnospira*; and whole raw almonds increased (*p* = 0.007) the relative abundance of *Dialister* (Table 3). There were no differences between control and almond butter. Almond consumption did not affect bacterial alpha diversity or beta diversity measures (Supplemental Figures S1–S4). 

## 4. Discussion

Herein, we report that almond consumption induced changes in the relative abundance of microbes present within the human gastrointestinal tract and that the degree of almond processing differentially impacted the relative abundance of fecal bacterial genera. With regard to specific microbes, the results from this randomized controlled dietary intervention that provided 42 g/d of almonds increased the relative abundance of *Clostridium* clusters IV and XIVa, including *Roseburia*, *Clostridium*, *Lachnospira* compared to the control diet period that was devoid of almonds. Comparisons between each of the four types of almonds (whole, whole roasted, roasted chopped, and butter) and control revealed that roasted chopped almonds increased the relative abundances of *Roseburia*, *Lachnospira*, and *Oscillospira*. Consumption of whole roasted almonds also increased the relative abundance of *Lachnospira*. There were no differences in the microbiota between the control group and the almond butter group. 

We previously reported that almond processing affected metabolizable energy, with whole natural, whole roasted, and roasted chopped almonds providing up to 25% less metabolizable energy than predicted by calculated Atwater values [19]. Herein, the secondary analyses conducted on the same fecal samples from that previous study provide evidence that food form also impacts the composition of the human intestinal microbiota. Indeed, consumption of roasted, chopped almonds increased the relative abundances of *Roseburia*, *Lachnospira*, and *Oscillospira* compared to control. Whole roasted almonds also increased the relative abundance of *Lachnospira*, however, they only tended to increase *Roseburia*. Similarly, there was only a trend for increased relative abundances of *Lachnospira* and *Roseburia* with whole, natural almonds. However, whole, natural almonds increased the relative abundance of *Dialister*. As the whole natural almonds were the hardest and generated a lower number of particles than chopped roasted and roasted whole almonds, this may have not only reduced the metabolizable energy but also the bioaccessibility of nutrients for microbial metabolism. Additional evidence for the relationship between metabolizable energy and the microbiota is provided through the comparisons between almond butter and control. Indeed, disruption of the plant cell wall by roasting and grinding the almonds into almond butter resulted in similar microbial profiles to that of the control period, which is consistent with our previously reported finding that the measured metabolizable energy of almond butter was not different from the calculated value [19]. Overall, these results indicate that roasting, which reduces the fracture force, hardness and subsequently particle size of almonds following mastication [29], as well as the metabolizable energy [19], are also relevant factors that affect the human gastrointestinal microbiota. 

Our findings are supported by previous clinical research that reported that up to 82 g/day of almonds did not affect alpha-diversity but did increased the abundance of several *Clostridium* spp. [30]. Similarly, a clinical trial assessing walnut consumption revealed that 42 g/day of walnuts increased *Roseburia*, *Clostridum* and *Dialister*, and reduced the relative abundances of *Bifidobacterium* [31]. Although there are not preclinical studies investigating the impact of almond consumption on gastrointestinal microbiota, our results are supported by a rodent study demonstrating that walnuts increased the relative abundances of Firmicutes, including *Clostridium* [32], *Roseburia*, and *Oscillospira* [33]. In vitro work has revealed that finely ground almonds increase butyrate concentrations [20]. *Roseburia* spp. produce high levels of butyrate in vitro, possess the butyryl coenzyme A (CoA): acetate CoA transferase, and may account for a significant proportion of butyrate-producing bacteria in the human colon [34,35,36]. Thus, the increase in the relative abundance of *Roseburia* following almond consumption may be important because reduced butyrate-producing bacteria has been linked to metabolic diseases including obesity [37], type 2 diabetes [2], and cardiovascular disease [38]. In addition, as our results revealed an increase in the relative abundance of *Roseburia* spp., we can hypothesize that these changes in the microbiota may have increased colonic butyrate concentrations in the study participants. However, additional human studies on almond consumption that measure fecal butyrate concentrations are needed to test this hypothesis as well as determine if changes in the gastrointestinal microbiota also contribute to metabolic health outcomes including adiposity, glycemia, and blood cholesterol concentrations. 

In addition to providing a good source of dietary fiber, almonds are also rich in unsaturated fatty acids. Unsaturated fatty acids, as opposed to saturated fatty acids, have been shown to have antimicrobial properties through the inhibition of bacterial fatty acid synthesis and growth [39], and also reduce microbial adhesion to mucus [40]. Thus, the increase in the relative abundance of *Roseburia* spp. following almond consumption in the present study may be related to their ability to metabolize fatty acids. Consumption of polyunsaturated fatty acids may modulate the composition of the gastrointestinal microbiome due to the differing abilities of bacteria to metabolize long-chain fatty acids. For example, in vitro studies have reported that *Roseburia* spp. are among the most active linoleic acid metabolizing bacteria in the human intestine [12,13,14]. 

Although the study participants’ age range was fairly broad, ranging from 32–78 years of age, 15 of the 18 study participates were 50 years of age or older. Therefore, the gastrointestinal microbiota characteristics of the participants in the current study would generally be reflective of a slightly older population. As the relative abundance of *Roseburia* spp. has been reported to be negatively associated with age [41,42], almond consumption may help delay age-related microbiota changes. However, as this study was fairly short-term (three weeks per diet period, 20 weeks total), additional longitudinal research on almond consumption and the microbiota in the context of aging is necessary. 

The strength of this study is the use of a complete feeding, randomized controlled, cross-over trial with washout periods between each treatment to assess the impact of almond processing on the human gastrointestinal microbiota. Study limitations include lack of bacterial fermentative end-products such as the short-chain fatty acids acetate, propionate, and butyrate. In summary, almond consumption and the form of the almonds differentially impacted the human gastrointestinal microbiota. Nutrients that might not be available to the body through digestion in the upper intestine due to the food matrix can become substrates for microbiota metabolism in the large intestine. In this study of different forms of almonds, when these nutrients enter the large intestine, these substrates alter the composition of the microbiota. These results suggest the gastrointestinal microbiota may contribute to the underlying mechanisms of the beneficial health effects of almond consumption. 

## Figures and Tables

**Table 1:** Baseline characteristics of the 10 male and eight female participants who consumed control or almond diets, each for three weeks.

Characteristics	Values ^1^	Range
Age, year	56.7 ± 10.2	32.7–72.4
BMI, kg/m^2^	29.7 ± 4.4	21.9–36.1
LDL cholesterol, mg/dL	121 ± 22.3	82.5–153
HDL cholesterol, mg/dL	57.9 ± 16.9	33.3–85.5
Triglycerides, mg/dL	102 ± 37.2	52.4–200
Glucose, mg/dL	94.8 ± 8.0	81.9–108

^1^ Values are means ± SD.

**Table 2:** Bacterial phyla and genera of human participants at the end of the control condition as compared to the end of the almond diet conditions.

Almond Consumption, % of Bacterial Sequences ^1^			
	Control (0 g/Day)	Almond (42 g/Day)	*p*-Value
Phyla and Genera			
Firmicutes	63.9 ± 1.99	65.6 ± 1.51	0.29
*Faecalibacterium*	4.39 ± 0.68	4.61 ± 0.64	0.47
*Coprococcus*	4.54 ± 0.54	4.70 ± 0.47	0.65
*Ruminococcus*	4.65 ± 0.70	4.16 ± 0.59	0.32
*Blautia*	2.09 ± 0.33	2.25 ± 0.30	0.41
*Dorea*	1.61 ± 0.28	1.69 ± 0.26	0.61
*Phascolarctobacterium* ^2^	0.23 ± 0.29	0.21 ± 0.28	0.67
*Roseburia* ^2^	0.47 ± 0.14	0.67 ± 0.13	0.03
*Lachnospira* ^2^	0.49 ± 0.15	0.71 ± 0.14	0.01
*Dialister*	0.42 ± 0.31	0.72 ± 0.29	0.05
*Clostridium* ^2^	0.42 ± 0.08	0.53 ± 0.07	0.04
*Streptococcus* ^2^	0.17 ± 0.18	0.17 ± 0.16	0.95
*Oscillospira*	0.54 ± 0.07	0.57 ± 0.06	0.56
Bacteroidetes	25.7 ± 2.13	26.7 ± 1.65	0.57
*Bacteroides*	19.2 ± 2.15	20.3 ± 1.87	0.41
*Parabacteroides*	1.31 ± 0.23	0.99 ± 0.20	0.02
*Prevotella* ^2^	0.03 ± 0.31	0.03 ± 0.30	0.24
Actinobacteria	5.45 ± 1.05	3.92 ± 0.91	0.03
*Bifidobacterium*	3.68 ± 0.85	2.48 ± 0.76	0.03
*Collinsella* ^2^	0.14 ± 0.24	0.13 ± 0.23	0.87
Verrucomicrobia	1.45 ± 0.34	1.10 ± 0.29	0.14
*Akkermansia*	1.45 ± 0.34	1.10 ± 0.29	0.14
Proteobacteria	2.34 ± 0.51	1.71 ± 0.39	0.15
	*n* = 18	*n* = 68	

^1^ Values are least-square means ± pooled SEM; *n* = 15–18 participants in a crossover design. ^2^ Values are mean log-normalized sequence abundances ± SE. Treatment effects of genera representing ≥0.5% of the total sequences were evaluated by using a mixed-model ANOVA (with fixed effects of period and treatment and a random effect of subject).

**Table 3:** Bacterial phyla and genera of human participants at the end of the control diet period and each of the almond diet periods, each for three weeks ^1^.

Almond Treatments, % of Sequences ^1^						
	Control 0 g/Day	Almond Butter 42 g/Day	Chopped Almonds 42 g/Day	Whole Roasted 42 g/Day	Whole Raw 42 g/Day	*p*-Value
PhylaandGenera						
Firmicutes	63.9 ± 1.99	64.2 ± 1.99	66.2 ± 2.13	66.2 ± 1.99	66.1 ± 2.03	0.68
*Faecalibacterium*	4.39 ± 0.68	4.62 ± 0.68	4.48 ± 0.70	4.40 ± 0.68	4.94 ± 0.68	0.63
*Coprococcus*	4.54 ± 0.54	4.60 ± 0.54	4.51 ± 0.56	5.06 ± 0.54	4.59 ± 0.54	0.73
*Ruminococcus*	4.65 ± 0.70	4.73 ± 0.69	3.70 ± 0.73	4.22 ± 0.69	3.88 ± 0.70	0.39
*Blautia*	2.09 ± 0.33	2.07 ± 0.33	2.36 ± 0.34	2.37 ± 0.33	2.24 ± 0.33	0.63
*Dorea*	1.61 ± 0.28	1.71 ± 0.28	1.67 ± 0.29	1.77 ± 0.28	1.58 ± 0.29	0.87
*Phascolarctobacterium* ^2^	0.23 ± 0.29	0.24 ± 0.29	0.23 ± 0.29	0.20 ± 0.29	0.19 ± 0.29	0.70
*Roseburia* ^2^	0.47 ± 0.14	0.48 ± 0.14	0.83 ± 0.14 *	0.73 ± 0.14 ^^^	0.73 ± 0.14 ^^^	<0.01
*Lachnospira* ^2^	0.49 ± 0.15	0.54 ± 0.15	0.80 ± 0.15 *	0.79 ± 0.15 *	0.76 ± 0.15 ^^^	0.02
*Dialister*	0.42 ± 0.31	0.44 ± 0.31	0.70 ± 0.32	0.74 ± 0.31	1.03 ± 0.31 *	0.01
*Clostridium* ^2^	0.42 ± 0.08	0.52 ± 0.08	0.54 ± 0.09	0.58 ± 0.08	0.48 ± 0.08	0.24
*Streptococcus* ^2^	0.17 ± 0.18	0.23 ± 0.18	0.17 ± 0.18	0.18 ± 0.18	0.11 ± 0.18	0.09
*Oscillospira*	0.54 ± 0.07	0.54 ± 0.07	0.75 ± 0.07 *	0.53 ± 0.07	0.51 ± 0.07	0.02
Bacteroidetes	25.7 ± 2.13	27.1 ± 2.14	26.5 ± 2.30	25.7 ± 2.14	27.6 ± 2.19	0.90
*Bacteroides*	19.2 ± 2.15	20.8 ± 2.15	20.4 ± 2.26	19.6 ± 2.15	20.6 ± 2.18	0.88
*Parabacteroides*	1.31 ± 0.23	1.07 ± 0.23	0.89 ± 0.23	1.03 ± 0.23	0.93 ± 0.23	0.13
*Prevotella* ^2^	0.03 ± 0.31	0.03 ± 0.31	0.04 ± 0.31	0.04 ± 0.31	0.03 ± 0.31	0.75
Actinobacteria	5.45 ± 1.05	4.51 ± 1.06	3.77 ± 1.11	4.09 ± 1.06	3.20 ± 1.07	0.16
*Bifidobacterium*	3.68 ± 0.85	2.68 ± 0.85	2.71 ± 0.89	2.53 ± 0.85	2.04 ± 0.86	0.19
*Collinsella* ^2^	0.14 ± 0.24	0.12 ± 0.24	0.12 ± 0.25	0.16 ± 0.24	0.13 ± 0.24	0.87
Verrucomicrobia	1.45 ± 0.34	1.16 ± 0.34	1.10 ± 0.36	1.25 ± 0.34	0.87 ± 0.35	0.44
*Akkermansia*	1.45 ± 0.34	1.16 ± 0.34	1.10 ± 0.36	1.25 ± 0.34	0.87 ± 0.35	0.44
Proteobacteria	2.34 ± 0.51	1.93 ± 0.51	1.88 ± 0.55	1.75 ± 0.51	1.29 ± 0.52	0.45
	*n* = 18	*n* = 18	*n* = 15	*n* = 18	*n* = 17	

^1^ Values are least-square means ± pooled SEM; *n* = 15–18 participants in a crossover design. ^2^ Values are mean log-normalized sequence abundances ± SE. Treatment effects of genera representing ≥0.5% of the total sequences were evaluated by using a mixed-model ANOVA with post-hoc Dunnet’s adjustments for multiple comparisons. Comparisons were made to the control condition. * *p* ≤ 0.05 for comparison between the respective treatment condition and control condition; ^ *p* ≤ 0.1 for comparison between the respective treatment condition and control condition.

## Supplementary Materials

The following are available online at http://www.mdpi.com/2072-6643/10/2/126/s1, Supplemental Figure S1: Phylogenetic Diversity, Supplemental Figure S2: Observed OTUs, Supplemental Figure S3: Unweighted Unifrac PCoA, Supplemental Figure S4: Weighted Unifrac PCoA.

## Abbreviations

Beltsville Human Nutrition Research Center (BHNRC), Body mass index (BMI), Principal coordinates analysis (PCoA), operational taxonomic unit (OTU), USDA (United States Department of Agriculture).
