# Supplementary material for: Almond Consumption and Processing Affects the Composition of the Gastrointestinal Microbiota of Healthy Adult Men and Women: A Randomized Controlled Trial
Source: Nutrients. 2018 Jan 26;10(2):126. doi: 10.3390/nu10020126 (PMC5852702; doi:10.3390/nu10020126)
Supplement: Supplementary file 1 [file nutrients-10-00126-s001.pdf]

Supplemental Figure 1: Phylogenetic Diversity

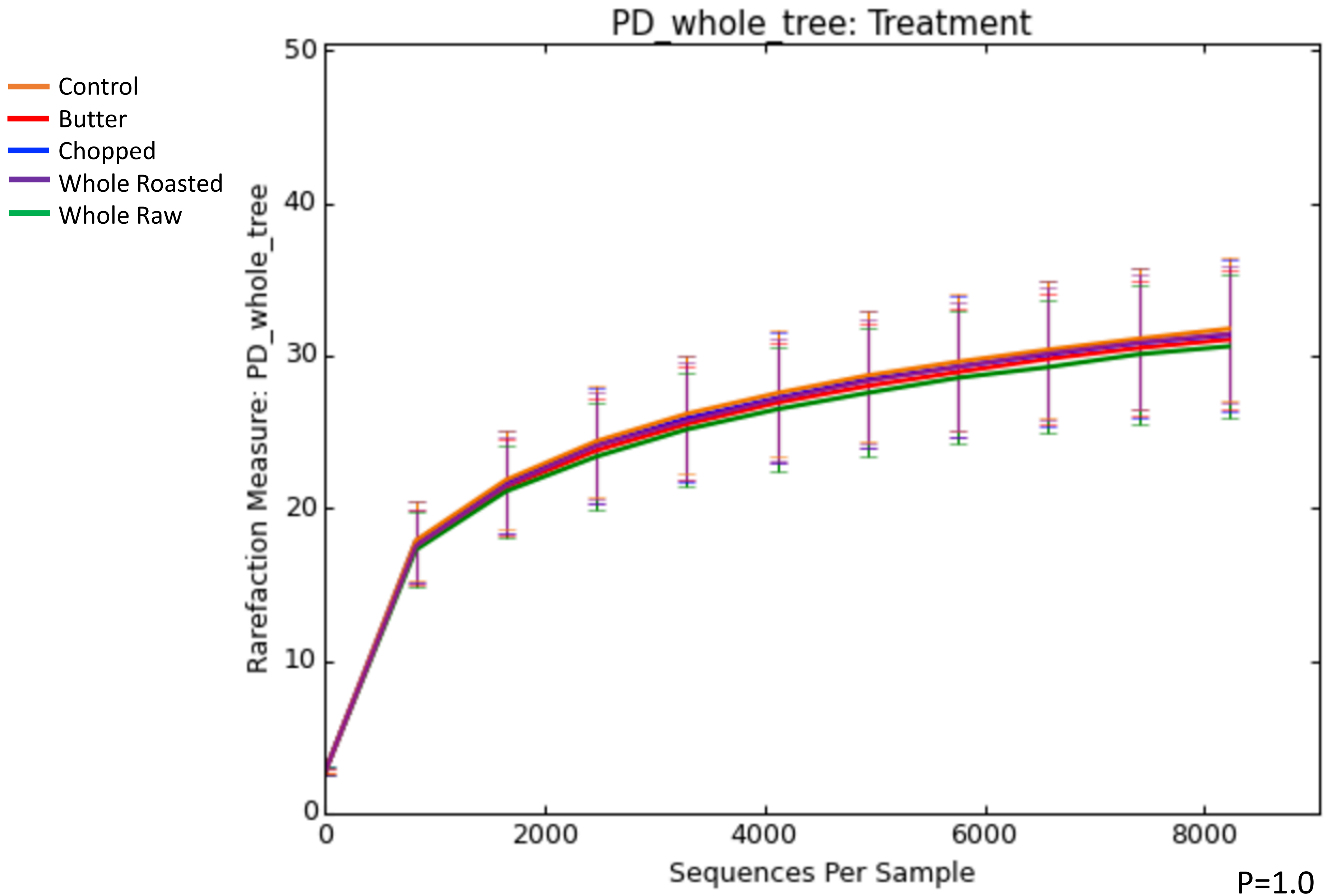

P=1.0

Supplemental Figure 2: Obeserved OTUs

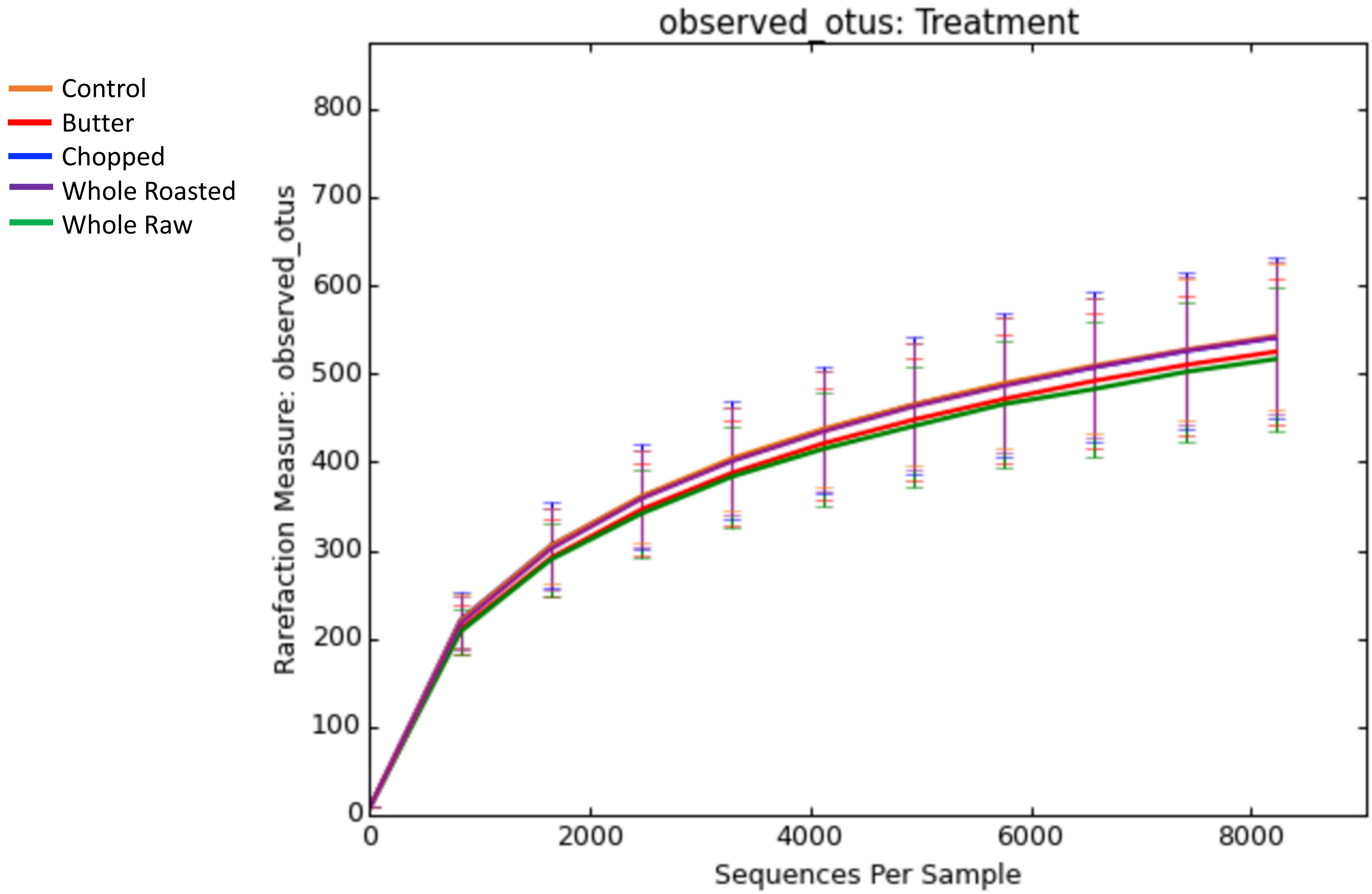

P=1.0

Supplemental Figure 3. Unweighted Unifrac PCoA

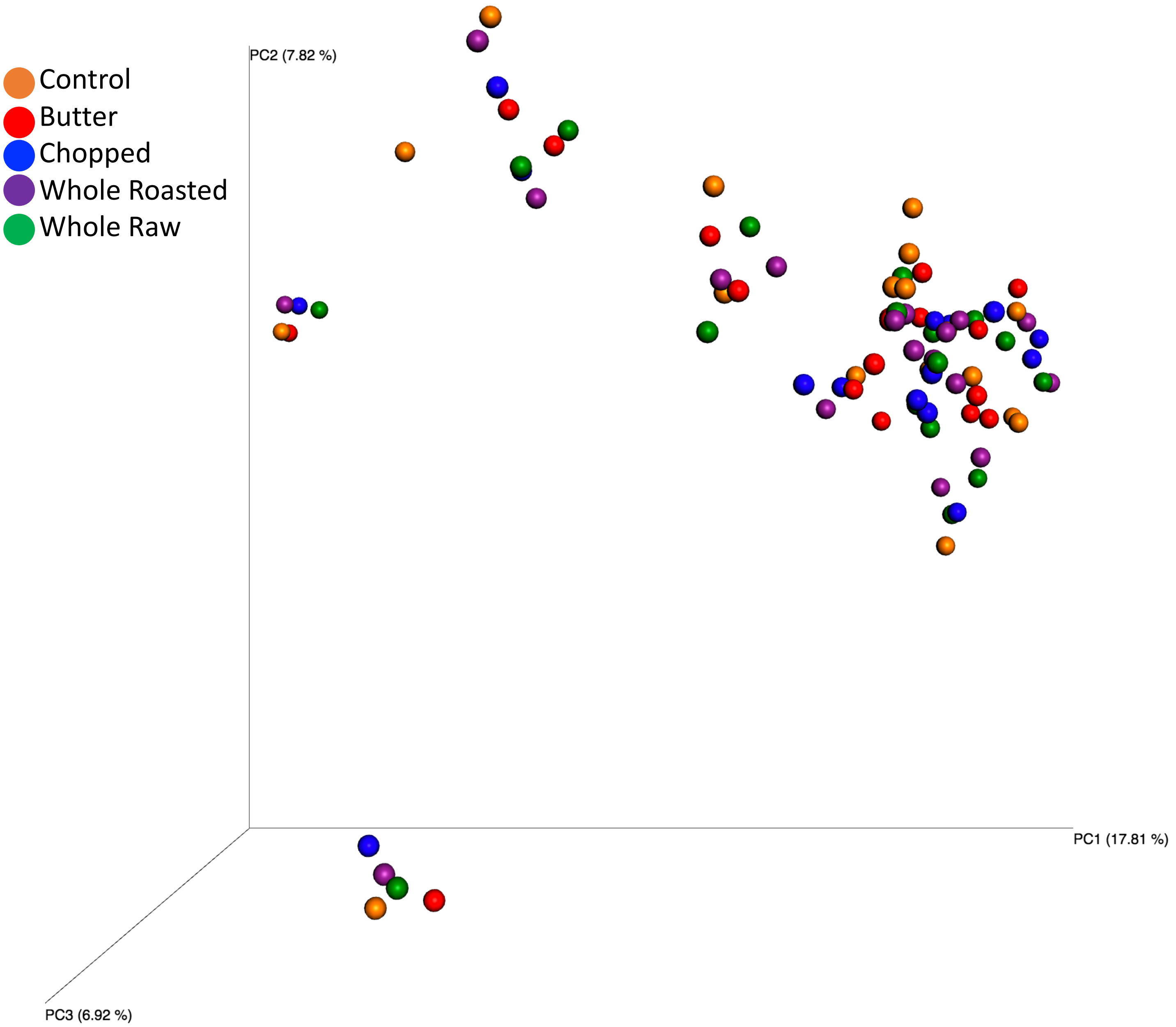

All within Treatment vs. All between Treatment t=3.5 p=0.001 Bonferroni-correct p-value =0.136

Supplemental Figure 4. Weighted Unifrac PCoA

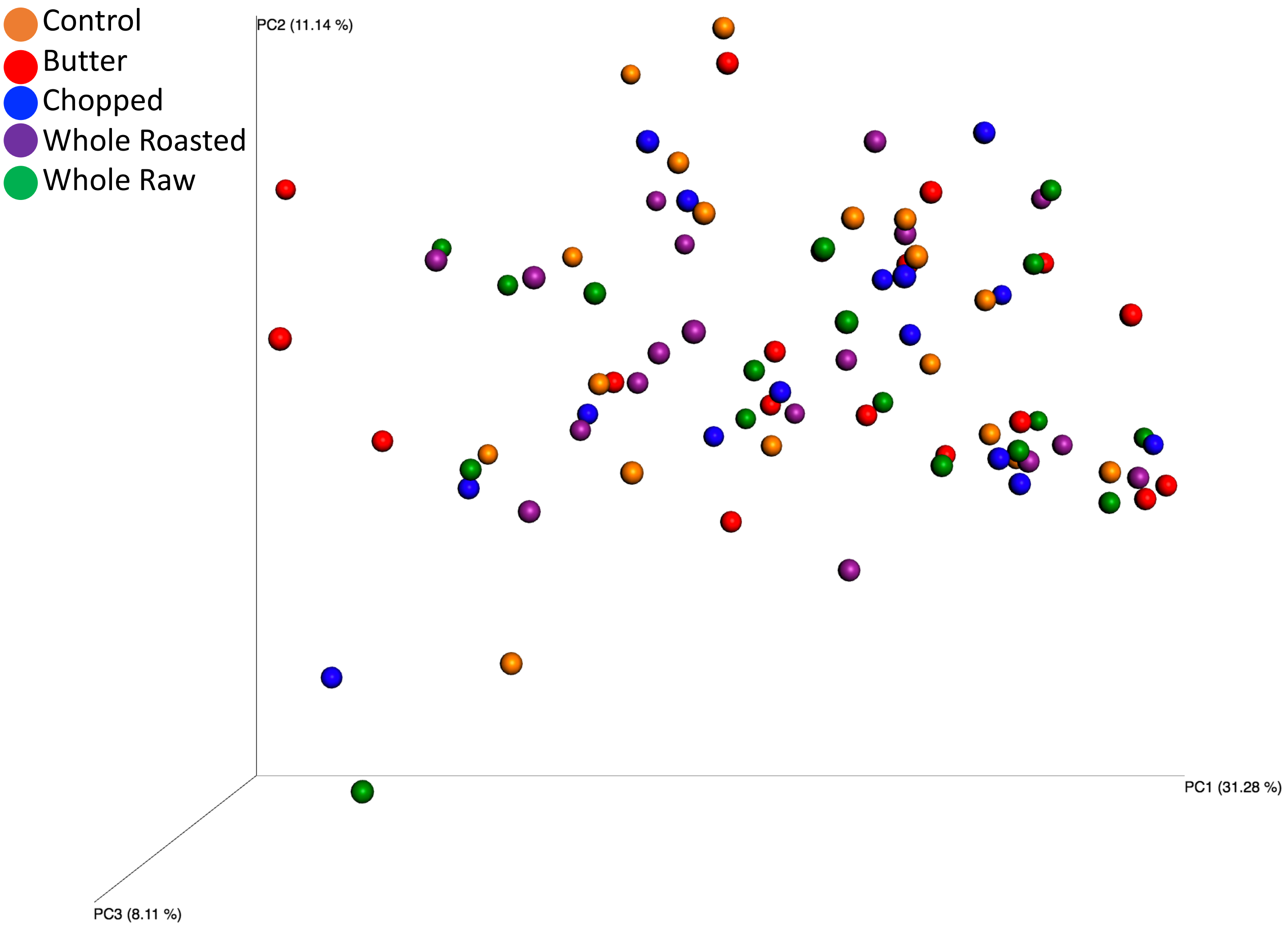

All within Treatment vs. All between Treatment t=2.1 p=0.03 Bonferroni-correct p-value=1.0
